# *Leuconostoc lactis* and *Staphylococcus nepalensis* Bacteremia, Japan

**DOI:** 10.3201/eid2609.191123

**Published:** 2020-09

**Authors:** Satoshi Hosoya, Satoshi Kutsuna, Daisuke Shiojiri, Saeko Tamura, Erina Isaka, Yuji Wakimoto, Hidetoshi Nomoto, Norio Ohmagari

**Affiliations:** National Center for Global Health and Medicine, Tokyo, Japan

**Keywords:** *Leuconostoc lactis*, *Staphylococcus nepalensis*, bacteremia, spontaneous esophageal rupture, bacteria, staphylococci, food safety, Japan

## Abstract

*Leuconostoc lactis* is a glycopeptide-resistant, gram-positive, facultative anaerobic coccus isolated from dairy products, whereas *Staphylococcus nepalensis* is coagulase-negative coccus that has not been identified as human pathogen. We report an instructive case of *L. lactis* and *S. nepalensis* bacteremia in a 71-year-old man who experienced Boerhaave syndrome after a meal.

*Leuconostoc lactis* is an intrinsically glycopeptide-resistant but ampicillin-susceptible, gram-positive, facultative anaerobic coccus (*1*) found in food products including dairy products, vegetables, and wine. *L. lactis* is a very rare pathogen associated with bloodstream infections (*2*). *Staphylococcus nepalensis* is a novobiocin-resistant coagulase-negative staphylococcus also found in food products, such as dry-cured ham and fish sauce, that has not been reported as a human pathogen (*3*–*5*). Neither *L. lactis* nor *S. nepalensis* is part of normal human bacterial flora (*2*,*3*).

A 71-year-old man with hypertension and hyperlipidemia sought care for upper abdominal pain and vomiting after a meal at his son’s restaurant. A computed tomography (CT) scan showed collapse of the lower esophagus wall and expansion of the mediastinum; medical staff diagnosed a spontaneous esophageal rupture and performed emergency surgery. Surgical findings demonstrated a 5 cm perforation of the lower esophagus with no rupture to the thoracic and abdominal cavity. The final diagnosis included Boerhaave syndrome, esophageal hiatus hernia, and mediastinitis. Two sets of blood culture taken on day 1 were positive for gram-positive cocci, which we identified by matrix-assisted laser desorption/ionization time-of-flight (MALDI-TOF) mass spectrometry as *L. lactis* in an aerobic bottle (10.7 h to culture) and an anaerobic bottle (13.3 h to culture) and *S. nepalensis* in 1 anaerobic bottle (24.3 h to culture). The 2 bacteria were indications of true bacteremia; therefore, we escalated ampicillin/sulbactam (treatment to piperacillin/tazobactam for *L. lactis* (Appendix Table 1) and initiated vancomycin treatment for *S. nepalensis* on day 3 after admission (Appendix Table 2). We measured MICs in the microdilution method using the MicroScan WalkAway 96 SI system (Beckman Coulter, https://www.beckmancoulter.com) with a MICrofast7J panel and determined the susceptibility of *L. lactis* according to Clinical and Laboratory Standards Institute (CLSI) guidelines (*6*). On day 7, we deescalated piperacillin/tazobactam to ampicillin/sulbactam, referring to the MICs, and we obtained follow-up sets of blood culture. The culture results were negative. We discontinued vancomycin by day 14 but maintained the ampicillin/sulbactam regimen. A follow-up CT scan on day 28 showed a subsiding mediastinal abscess. Moreover, a pathological examination of the surgical biopsy demonstrated no esophageal cancer. On the basis of the clinical course of the disease, we strongly suspected a breakthrough of *L. lactis* and *S. nepalensis* through the ruptured esophagus into the bloodstream. To prove this relationship, we obtained permission from the patient’s son to analyze samples of the food products his father consumed, including cheese, dry-cured ham, sauerkraut, pizza margherita, bianchetti (pasta with boiled young sardines), and red and white wine. We cultured samples from these products on blood agar medium; colonies of *L. lactis*, confirmed by MALDI-TOF mass spectrometry, were derived from cheese samples (Figure).

**Figure:**
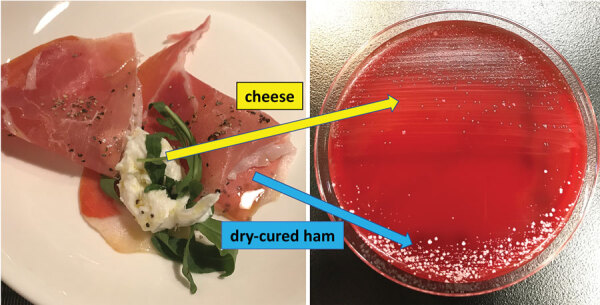
Culture of cheese and dry-cured ham on blood agar medium from investigation of patient with *Leuconostoc lactis* and *Staphylococcus nepalensis* bacteremia, Japan. The colonies, cultured from cheese, were identified as *L. lactis* by matrix-assisted laser desorption/ionization time-of-flight mass spectrometry mass spectrometry; however, the colonies derived from dry-cured ham were identified as *S. equorum* and *S. xylosus* but not as *S. nepalensis*.

Approximately 20 cases of *L. lactis* bacteremia have been reported (*1*), mostly in immunosuppressed patients with malignancy including leukemia, diabetes, or impaired skin barrier function due to central venous catheter. Several entry routes to the bloodstream have been hypothesized, including the digestive tract or the skin in catheter-related bloodstream infections, or as a result of microbial substitution due to glycopeptide administration; however, no entry point has been definitively identified (*1*,*2*,*7**,**8*). In addition, *L. lactis* bacteremia caused by gastrointestinal tract perforation had not been reported. We concluded that *L. lactis* colonized cheese and entered the bloodstream through a perforation of the lower esophagus, and we were able to demonstrate that *L. lactis* can enter the bloodstream through a rupture of the digestive tract. Based on our findings, we may advise screening for gastrointestinal diseases, such as ulcer, perforation, and malignancy, in patients with *L. lactis* bacteremia.

*S. nepalensis* has not previously been reported as a human pathogen, nor has its pathogenicity been described. Because the results of the food sample cultures identified other coagulase-negative *Staphylococci* bacteria (*S. equorum* and *S. xylosus*), rather than *S. nepalensis*, from the dry-cured ham colonies, we could not conclusively demonstrate the entry of *S. nepalensis* to the bloodstream. Moreover, contamination with *S. nepalensis* was possible; only 1 anaerobic bottle of blood culture taken at admission was positive. However, because *S. nepalensis* is not normally found in the human microbial flora but is a part of the predominant flora in dry-cured ham together with *S. equorum* and *S. xylosus* (*4*), we suspect that the *S. nepalensis* bacteremia diagnosis was correct.

In conclusion, we demonstrate the point of entry for *L. lactis* into the human bloodstream and show results implying that *L. lactis* can be a pathogen of bacteremia, as previous reports have shown (*1*,*2*,*7**,**8*). Our report is also a suspected case of *S. nepalensis* bacteremia; further investigation is needed for confirmation.

Appendix Additional information about *Leuconostoc lactis* and *Staphylococcus nepalensis* bacteremia in a patient with spontaneous esophageal rupture.
