# Supplementary material for: Leuconostoc lactis and Staphylococcus nepalensis Bacteremia, Japan
Source: Emerg Infect Dis. 2020 Sep;26(9):2283–5. doi: 10.3201/eid2609.191123 (PMC7454056; doi:10.3201/eid2609.191123)
Supplement: Appendix — Additional information about Leuconostoc lactis and Staphylococcus nepalensis bacteremia in a patient with spontaneous esophageal rupture. [file 19-1123-Techapp-s1.pdf]

Article DOI: https://doi.org/10.3201/eid2609.191123

# *Leuconostoc lactis* and *Staphylococcus nepalensis* Bacteremia, Japan

## Appendix

**Appendix Table 1.** Antimicrobial susceptibility of *L. lactis* according to Clinical and Laboratory Standards Institute (CLSI) criteria*

| Antimicrobial drug | MIC | Susceptibility |
|---|---|---|
| PCG | 0.5 | S |
| AMPC | 1 | S |
| AMPC/CVA | ≥8 | – |
| CTM | ≥8 | – |
| CDTR-PI | ≥2 | – |
| CTX | ≥8 | – |
| CTRX | ≥8 | – |
| CZOP | ≥8 | – |
| CFPM | ≥4 | – |
| MEPM | ≥4 | – |
| EM | ≤0.12 | – |
| AZM | ≤0.12 | – |
| CLDM | ≤0.12 | – |
| MINO | 1 | S |
| CP | ≤4 | S |
| VCM | ≥2 | – |
| LVFX | 2 | – |
| ST | ≤0.5 | – |
| RFP | ≥8 | – |

*AMPC, ampicillin; AMPC/CVA, amoxicillin/clavulanate; AZM, azithromycin; CDTR-PI, cefditoren pivoxil; CFPM, cefepime; CLDM, clindamycin; CP, chloramphenicol; CTM, clarithromycin; CTRX, ceftriaxone; CTX, cefotaxime; CZOP, cefozopran; EM, erythromycin; LVFX, levofloxacin; MEPM, meropenem; MIC, minimal inhibitory concentration; MINO, minocycline; PCG, penicillin G; RFP, rifampin; S, susceptible; ST, sulfamethoxazole-trimethoprim; VCM, vancomycin.

**Appendix Table 2.** Antimicrobial susceptibility of *S. nepalensis* according to Clinical and Laboratory Standards Institute (CLSI) criteria

| Antimicrobial drug | MIC | Susceptibility |
|---|---|---|
| PCG | $\leq$0.06 | R |
| MPIPC | 0.5 | S |
| ABPC | $\leq$1 | R |
| ABPC/SBT | $\leq$2 | S |
| CEZ | $\leq$1 | S |
| CMZ | $\leq$4 | S |
| CFX | $\leq$4 | – |
| IPM/CS | $\leq$1 | S |
| GM | $\leq$1 | S |
| ABK | $\leq$1 | – |
| EM | $\geq$8 | R |
| CLDM | 2 | I |
| MINO | $\leq$1 | S |
| VCM | 1 | S |
| TEIC | $\leq$1 | S |
| DAP | $\leq$0.25 | S |
| LVFX | $\leq$0.5 | S |
| FOM | $\leq$4 | S |
| ST | $\leq$0.5 | S |
| RFP | $\leq$0.5 | S |
| LZD | 1 | S |
| MUP | $\leq$256 | S |

*ABK, arbekacin; ABPC/SBT, ampicillin/sulbactam; CEZ, cefazoline; CFX, cefotiam; CLDM, clindamycin; CMZ, cefmetazole; DAP, daptomycin; EM, erythromycin; FOM, fosfomycin; GM, gentamicin; I, intermediate; IPM/CS, imipenem/cilastatin; LVFX, levofloxacin; LZD, linezolid; MIC, minimal inhibitory concentration; MINO, minocycline; MPIPC, oxacillin; MUP, mupirocin; PCG, penicillin G; R, resistant; RFP, rifampin; S, susceptible; ST, sulfamethoxazole-trimethoprim; TEIC, teicoplanin; VCM, vancomycin.
